# Toward a framework of muscularity-oriented disordered eating

**DOI:** 10.1186/2050-2974-1-S1-O50

**Published:** 2013-11-14

**Authors:** Scott Griffiths, Stuart Murray, Stephen Touyz

**Affiliations:** 1 University of Sydney, Australia; 2 Redleaf Practice, Australia

## 

Sociocultural forces promoting a thin ideal body for women have shaped our understanding of problematic eating attitudes and behaviours throughout the 20th century, giving rise to the modern, thinness-oriented definition of disordered eating. More recent cultural expectations of a lean and muscular body, particularly for males, have been named as likely contributors to the growing prevalence of muscularity-focused body image disturbance amongst boys and men. However, disordered eating in the pursuit of muscularity appears in stark contrast to that which is motivated by the drive for thinness, suggesting that a new disordered eating framework specific to muscularity is needed. We define muscularity-oriented disordered eating and propose a framework constituting five major components, including body image concerns, eating, nutritional supplements, appearance and performance enhancing drugs, and exercise. We critically analyse the nature, prevalence and effects of these facets of muscularity-oriented disordered eating, and suggest improvements and directions for future research. Intentions of this framework are to inform clinicians engaged in the treatment of muscularity-related body image and eating disturbance, and to guide future empirical research and interventions in this field.

This abstract was presented in the **Body Image** stream of the 2013 ANZAED Conference.

